# Safety and Effectiveness of Placental Connective Tissue Matrix Allograft Versus Corticosteroid Injection in Adhesive Capsulitis: A Double-Blinded Prospective Randomized Study

**DOI:** 10.2106/JBJS.OA.26.00080

**Published:** 2026-08-27

**Authors:** Olivia C. Nembhard, Janice M. Pogoda, James M. Helm, James M. Gregory, Jessica Waters, Ajai Sambasivan, Evan Meeks, Paul Shupe, Eric F. Berkman

**Affiliations:** 1 Department of Orthopedic Surgery, University of Texas Health Science Center at Houston, Houston, Texas

## Abstract

**Background:**

Adhesive capsulitis causes significant pain and functional limitation, with limited effective treatment options. Current standard-of-care treatments, such as corticosteroid injections, may offer short-term symptom relief but are associated with limited long-term efficacy and potential side effects. Regenerative therapies using extracellular matrix–based products derived from placental tissue have shown promise in treating musculoskeletal injuries due to their anti-inflammatory and tissue-healing properties. Clinical data supporting their use in adhesive capsulitis remains limited. The objective of this study was to compare the safety and effectiveness of a flowable placental connective tissue matrix allograft (PCTMA) vs. corticosteroid injection for adhesive capsulitis.

**Hypothesis:**

We hypothesized that intra-articular injection of a placental connective tissue allograft would result in superior clinical outcomes compared with corticosteroid injection for adhesive capsulitis of the shoulder.

**Study Design:**

Randomized controlled clinical trial.

**Methods:**

This double-blinded prospective study randomized patients with adhesive capsulitis to receive either intra-articular PCTMA (1 cc) with lidocaine (study group, n = 25) or corticosteroid injection with triamcinolone 20 mg and lidocaine (control group, n = 27). Inclusion criteria included ages 18 to 75 years, clinical diagnosis of adhesive capsulitis, shoulder pain >6 weeks, and MRI confirmation without other shoulder pathology. The primary end point was change from baseline in Shoulder Pain and Disability Index (SPADI) score. Secondary end points included Visual Analog Scale (VAS), American Shoulder and Elbow Surgeons (ASES) score, and passive range of motion. Assessments occurred at 4, 12, and 24 weeks postinjection.

**Results:**

Both groups improved over time. At 24 weeks, mean SPADI improvement was similar between groups [PCTMA: −40.4 (SD 18.81) vs. corticosteroid: −37.7 (SD 30.12)] (p = 0.73), but post hoc responder analyses favored PCTMA: SPADI responders 88% vs. 63% (p = 0.04), VAS responders 80% vs. 56% (p = 0.06), and ASES responders 88% vs. 56% (p = 0.01). One serious adverse event unrelated to the study intervention was reported.

**Conclusion:**

Flowable intra-articular PCTMA demonstrated a favorable safety profile and comparable improvements in pain and disability to corticosteroid injection at 24 weeks. A post hoc responder analysis favored PCTMA, suggesting potential for more sustained clinical benefit warranting further investigation.

**Clinical Relevance:**

This study provides the first randomized controlled comparison of PCTMA vs. corticosteroid injection for adhesive capsulitis, offering preliminary evidence to guide treatment selection and informing the design of future, larger-scale trials investigating biologic therapies for this condition.

**Trial Registration:**

The authors confirm that the protocol was finalized before patient enrollment. Clinical trial registration (NCT05844930) was completed in April 2023.

**Level of Evidence:**

Therapeutic Level II.See Instructions for Authors for a complete description of levels of evidence.

## Introduction

Adhesive capsulitis, or frozen shoulder, is a common cause of shoulder pain and stiffness, affecting approximately 2% to 5% of the general population^1,2^. Characterized by progressive fibrosis and contracture of the glenohumeral joint capsule, the disease often results in pain and restricted motion that can persist for months to years^1^. Traditional interventions such as physical therapy, nonsteroidal anti-inflammatory drugs, and corticosteroid injections can provide short-term symptomatic relief; however, they often fail to alter the long-term natural course of the disease^2,3^.

Recent attention has turned toward biologic regenerative therapies, particularly extracellular matrix (ECM) allograft products derived from placental tissue. Placental-derived connective tissue matrices contain structural proteins, growth factors, and anti-inflammatory cytokines known to promote tissue repair and modulate fibrosis^4-6^. Preclinical and clinical studies suggest that ECM-derived injectables are safe and may support soft-tissue healing and pain reduction across musculoskeletal conditions^7-9^.

The aim of this study was to evaluate the safety and clinical efficacy of a flowable placental connective tissue matrix allograft (ActiveMatrix®, Skye Biologics Holdings, LLC) compared with corticosteroid injection in patients with adhesive capsulitis. We hypothesized that PCTMA would demonstrate comparable or superior clinical outcomes without increased adverse events.

## Methods

This study was a prospective, double-blinded, randomized controlled trial conducted at a single center. The study was reviewed and approved by an Institutional Review Board (Protocol No. Pro00054360). This trial was registered at ClinicalTrials.gov (Identifier: NCT05844930) in April 2023. The trial protocol is available through the ClinicalTrials.gov. We disclose that patient enrollment commenced before April 2023 in January 2023. However, the study protocol and all primary and secondary end points were finalized and locked before the enrollment of the first patient. All investigators remained blinded to the data, and no interim analyses were performed before the formal registration date. This trial was sponsored by the manufacturer of the PCTMA product used in this randomized controlled trial.

Written informed consent was obtained from all participants before enrollment. The estimated sample size was 25 participants per group, assuming an anticipated attrition rate of 20%. Participants were enrolled between January 2023 and September 2024. Follow-up study visits occurred between January 2023 and February 2025. Eligible patients were adults aged 18 to 75 years with clinical and MRI-confirmed unilateral adhesive capsulitis defined by the following: painful shoulder stiffness for greater than 6 weeks, passive range of motion (PROM) limitation >20° compared with the contralateral side in at least 2 planes, and MRI confirmation excluding rotator cuff tear, osteoarthritis, or other intra-articular pathology. Exclusion criteria included prior shoulder surgery; systemic inflammatory disease; infection; poorly controlled diabetes mellitus, as defined by HbA1c > 8 mmol/L and glucose > 200 mg/dL; history of cerebrovascular accident such as stroke or transient ischemic attack; blood dyscrasias; participants of childbearing potential who test positive for pregnancy; or corticosteroid injection within 3 months.

Participants were randomized 1:1 to receive either a single intra-articular corticosteroid injection (triamcinolone 20 mg + lidocaine 5 cc) or placental connective tissue allograft (ActiveMatrix 1 cc + lidocaine 5 cc). The use of PCTMA as an intra-articular injection for adhesive capsulitis represents an off-label application. The investigational product was supplied by the manufacturer. The steroid dosage of 20 mg triamcinolone was selected based on a randomized controlled trial demonstrating equivalent efficacy between 20 and 40 mg for adhesive capsulitis, with the lower dose chosen to minimize potential steroid-related side effects^10^. Participants were randomized using opaque envelopes containing the treatment allocation. The envelopes were prepared before enrollment by a member of the research team, sealed, and shuffled to ensure random assignment. Envelopes were opened only after a participant had provided informed consent and was enrolled in the study. All injections were performed by a single physician under ultrasound guidance by a posterior approach. Ultrasound images documenting needle placement were captured to confirm consistent intra-articular injection location across participants. Patients were blinded to treatment allocation. Patient-reported outcomes were collected through self-administered electronic questionnaires completed independently by participants at each visit. PROM assessments were performed by the treating surgeon, who remained blinded to treatment allocation throughout the study. Participants were instructed to avoid strenuous activity for 48 hours, followed by a standardized home rehabilitation program using a digital physical therapy platform with a nonoperative frozen shoulder protocol. No additional injections were permitted during the 24-week follow-up.

Assessments were conducted at baseline and 4, 12, and 24 weeks postinjection. Patients who failed to respond to follow-up communication after 2 missed visits were classified as lost to follow-up. Before classification as lost to follow-up, attempts to contact patients included multiple phone calls from both the research coordinator and the clinic's medical assistant, as well as written electronic communication requesting scheduling of outstanding visits. Adverse events were documented and reported according to protocol definitions.

### Statistical Methods

Analyses of efficacy end points included all randomized patients. The primary outcome was change from baseline to 24 weeks in Shoulder Pain and Disability Index (SPADI) score. Secondary outcomes were change from baseline to 24 weeks in Visual Analog Scale (VAS) for pain, American Shoulder and Elbow Surgeons (ASES) score, and PROM in flexion, abduction, and external and internal rotation. Treatment-emergent adverse events were evaluated in patients treated with either corticosteroid injection or PCTMA.

Changes from baseline were calculated as the observed value at the given visit minus the value at baseline and were summarized using means and SDs. Baseline was defined as the last observation before treatment. Differences between treatment groups were tested using Satterthwaite *t*-tests. Differences within treatment groups were tested using Student’s *t*-tests.

Response was assessed in post hoc analyses, with patients who discontinued early from the study counted as nonresponders. Response was defined as improvement from baseline greater than or equal to the minimal clinically important difference (MCID). The MCID for SPADI,^1,9^ VAS,^11,12^ ASES,^12^ and PROM^13^ was defined as 13, 1, 8.2, and 10 points, respectively. Differences in response rates at each visit were analyzed using either chi square tests of association or Fisher’s exact tests (where expected counts were less than 5).

All statistical analyses were performed using SAS^®^ software version 9.4 (SAS Institute, Inc., Cary, NC, USA). There were no adjustments for multiplicity and no imputations performed other than as noted above for post hoc analyses.

## Results

**Fig. 1:**
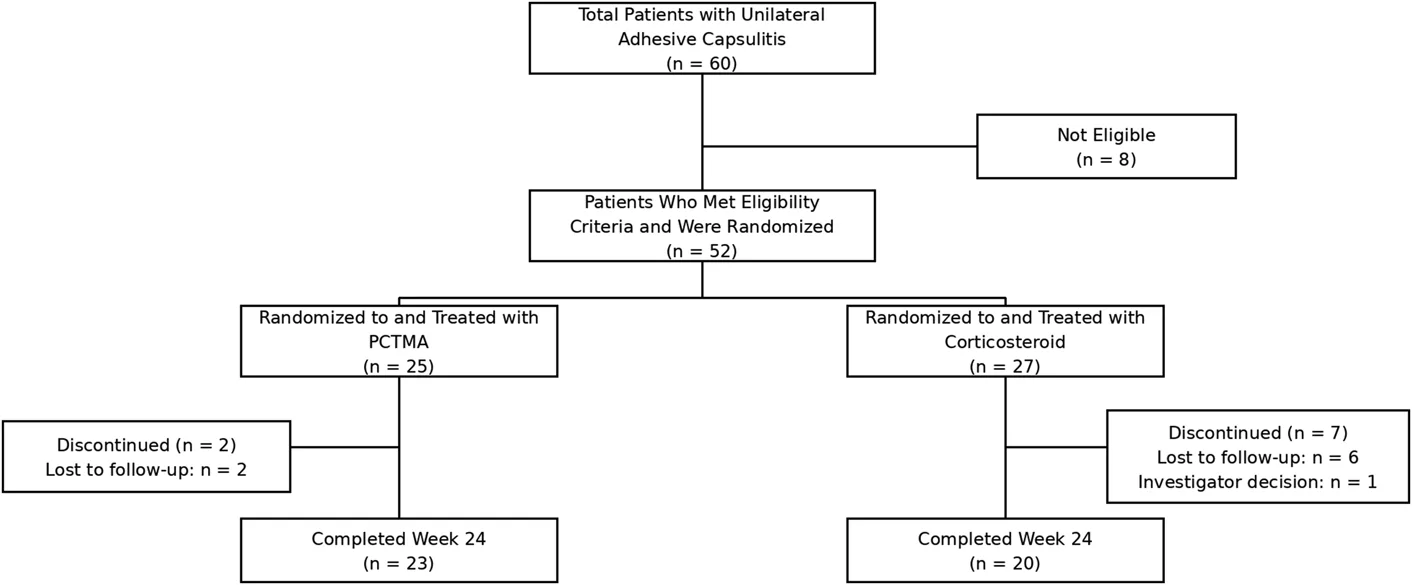
Consolidated Standards of Reporting Trials flow diagram. PCTMA = placental connective tissue matrix allograft.

Of 60 screened patients, 52 met inclusion criteria and were randomized (PCTMA, n = 25; corticosteroid, n = 27). Two patients (8%) were lost to follow-up in the PCTMA group and 6 (22%) in the corticosteroid group. One patient (4%) in the corticosteroid group was discontinued due to investigator decision. This patient presented at a follow-up visit with severe pain that had worsened from baseline and was withdrawn from the study by the principal investigator to pursue more appropriate pain management. Baseline characteristics were descriptively similar between groups (Table I). Patient enrollment, allocation, follow-up, and analysis are summarized in Figure 1.

**Table I:** Baseline Demographics and Clinical Characteristics of Randomized Participants

Variable	PCTMA (n = 25)	Corticosteroid (n = 27)
Age (years), mean (SD)	59 (10.0)	54 (9.3)
Female, n (%)	13 (52.0)	17 (63.0)
Duration of pain, n (%)		
< 6 months	14 (56.0)	13 (48.1)
6 months to 1 year	10 (40.0)	8 (29.6)
≥ 1 year	1 (4.0)	6 (22.2)
Baseline SPADI, mean (SD)	57.4 (21.46)	59.1 (23.82)
Baseline ASES, mean (SD)	40.6 (18.24)	39.8 (21.19)

ASES = American Shoulder and Elbow Surgeons, PCTMA = placental connective tissue matrix allograft, and SPADI = Shoulder Pain and Disability Index.

**Fig. 2:**
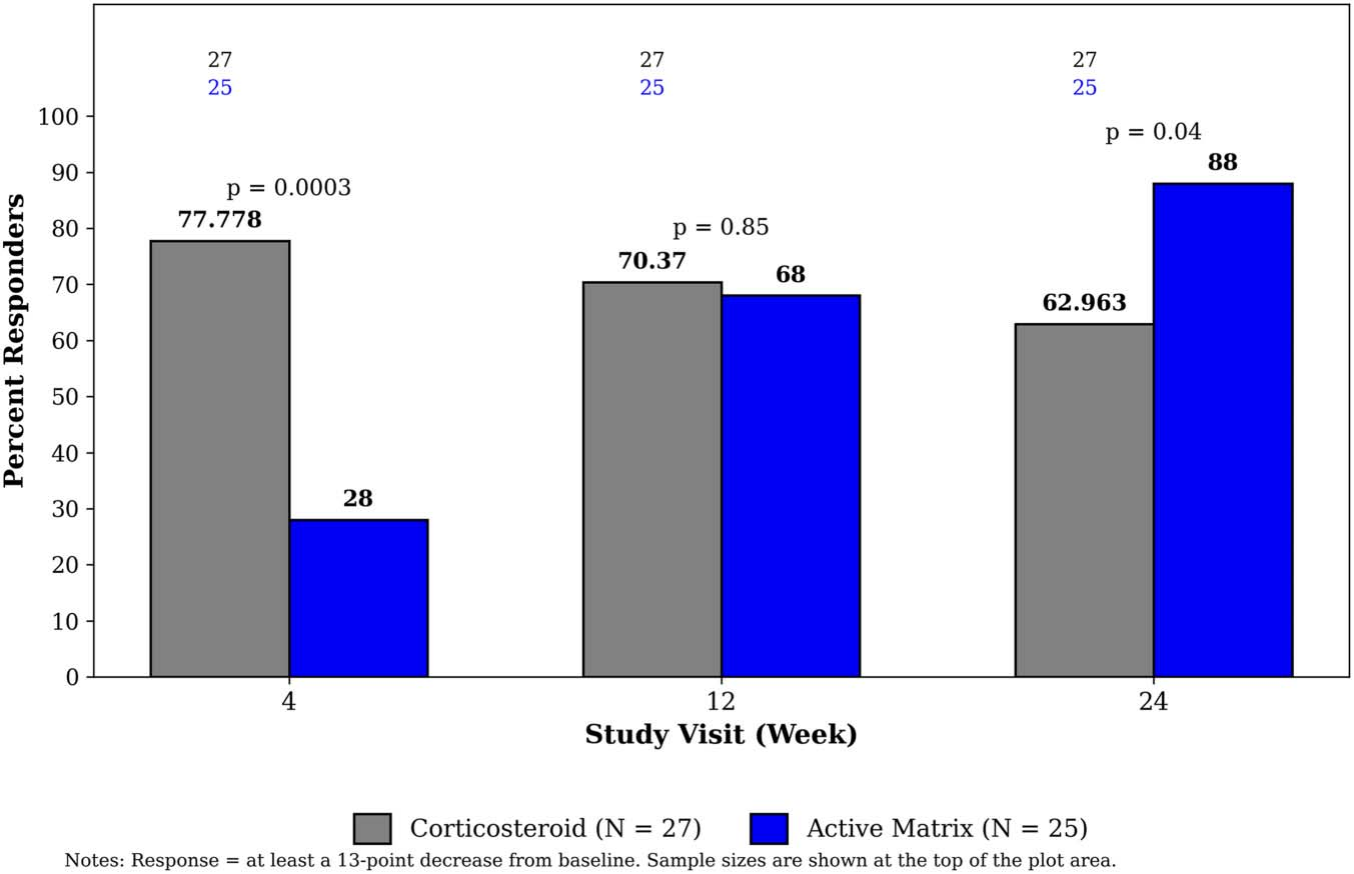
Response based on Shoulder Pain and Disability Index total score by treatment group. Full analysis set, missing values imputed as nonresponse.

The mean (SD) SPADI improvement from baseline to Week 24 was −40.4 (18.81) for PCTMA and −37.7 (30.12) for corticosteroid (p = 0.73). Both groups achieved significant within-group improvement from baseline to Week 24 (p < 0.0001). The proportion of SPADI responders at week 24 favored PCTMA (88% vs. 63%, p = 0.04) (Fig. 2).

VAS and ASES scores descriptively improved in both groups (Table II). The PCTMA group showed progressive improvement from 4 to 24 weeks, whereas corticosteroid benefits plateaued after week 4. For both VAS and ASES, responder rates were higher in PCTMA at week 24 (VAS 80% vs. 56%, p = 0.06; ASES 88% vs. 56%, p = 0.01). PROM internal rotation improvements were consistently greater in the PCTMA group across all time points. One serious adverse event (myocardial infarction) occurred in a participant randomized to the corticosteroid group and was unrelated to the study intervention. No treatment-related conditions were reported.

**Table II:** Change from Baseline in Clinical Outcomes at 4, 12, and 24 weeks Among Randomized Participants

Outcome	Visit	PCTMA	Corticosteroid	p
SPADI	4 weeks			
	n	25	23	
	Mean (SD)	−9.5 (17.69)	−37.8 (22.49)	<0.0001
SPADI	12 weeks			
	n	23	23	
	Mean (SD)	−27.7 (17.69)	−37.8 (22.49)	0.14
SPADI	24 weeks			
	n	23	20	
	Mean (SD)	−40.4 (18.81)	−37.7 (30.12)	0.73
ASES	24 weeks			
	n	23	20	
	Mean (SD)	40.9 (17.43)	35.3 (31.57)	0.49
VAS	24 weeks			
	n	23	20	
	Mean (SD)	−4.8 (2.40)	−3.9 (3.19)	0.32

ASES = American Shoulder and Elbow Surgeons, PCTMA = placental connective tissue matrix allograft, SPADI = Shoulder Pain and Disability Index, and VAS = Visual Analog Scale.

## Discussion

This randomized controlled trial demonstrates that intra-articular PCTMA provides comparable pain and disability improvement to corticosteroid injection. In a post hoc analysis in which patients who discontinued early from the study counted as nonresponders, PCTMA yielded better clinical responder rates at 24 weeks. Both treatments were safe and well-tolerated.

The early benefit seen with corticosteroid aligns with existing literature demonstrating rapid, transient symptom relief with limited durability^7,9^. Conversely, biologic ECM-based injectables may act through modulation of inflammation and promotion of extracellular remodeling, leading to progressive and sustained improvement, likely due to their bioactive matrix composition containing anti-inflammatory and regenerative cytokines^5,6,8^. The higher proportion of responders in the PCTMA group in the post hoc analysis supports the hypothesis that biologic therapies may more directly address the underlying pathophysiology of adhesive capsulitis rather than solely suppressing inflammation, as suggested by sustained improvements in pain and function reported with mechanical intervention such as ultrasound-guided hydrodilation^14^. The mechanical and biological interventions that target the joint capsule may improve long-term outcomes beyond symptom suppression.

Preclinical data have shown that ECM-based grafts contain collagens, glycosaminoglycans, and bioactive peptides that modulate macrophage polarization and can modulate transforming growth factor-β–driven fibrosis, enhance collagen organization and support endogenous tissue repair^3,4,8,15,16^. These properties may attenuate the chronic capsular inflammation and fibrosis that characterize adhesive capsulitis, potentially restoring joint compliance in a manner distinct than corticosteroid alone. Our post hoc findings are consistent with these mechanisms and reinforce prior pilot data showing favorable responder rates^17,18^.

This study has several limitations. The single-center design and modest sample size may limit generalizability. Objective imaging or biochemical markers of inflammation were not evaluated. Follow-up was limited to 24 weeks, precluding assessment of long-term durability. While patients and the treating surgeon performing PROM assessments were blinded, the absence of a saline placebo arm limits mechanistic differentiation between biologic and injection effects. Multivariable analysis to control for potential confounders, such as the use of pain medication and baseline symptom duration, was not performed due to small sample size and, thus, the inability to properly evaluate interactive effects. However, at week 24, no patients reported the use of strong pain medication and a higher proportion of patients in the corticosteroid group reported any use of pain medication; thus, it seems unlikely that results were confounded by pain medication use. Of note, a higher proportion of patients in the corticosteroid group reported pain duration greater than 1 year, which may have contributed to a lower response rate in this group. There were no adjustments for multiple tests of inference to control type 1 error. The only statistically significant finding favoring PCTMA was through a post hoc analysis in which patients who discontinued early from the study were counted as nonresponders. Future studies with larger populations, extended follow-up, and prespecified efficacy end points with strategies for confounders and/or effect modifiers as well as for multiplicity adjustment informed by this study are warranted.

## Conclusion

Intra-articular flowable PCTMA demonstrated a favorable safety profile and comparable improvements in pain and disability to corticosteroid injection at 24 weeks. A post hoc responder analysis favored PCTMA, suggesting potential for more sustained clinical benefit. The preliminary findings in this study support further investigation of placental-derived biologics for adhesive capsulitis in larger, prospective trials.

## Funding

This study was sponsored by Skye Biologics, LLC. The sponsor provided the investigational product used in the study and financial support to The University of Texas for study-related costs, including study startup, operational expenses, statistical analysis support, and study closeout activities.

## Conflict of Interest

One or more of the authors has declared a potential conflict of interest. Janice Pogoda, PhD, reports receiving consulting fees from Skye Biologics, LLC for statistical support related to this study. The remaining authors declare no potential conflicts of interest.

**What Is Known About the Subject**

- Adhesive capsulitis is commonly treated with corticosteroid injections to reduce pain and improve shoulder mobility.^1^
- While corticosteroid injections can provide short-term symptom relief, their effects may diminish over time, and recurrence of symptoms is common.^2,3^
- Biologic therapies, including connective tissue–derived products, are being investigated as potential alternatives to improve outcomes in adhesive capsulitis.^4-6^

**What This Study Adds to Existing Knowledge**

- To our knowledge, this is the first randomized controlled trial examining intra-articular PCTMA specifically for the treatment of adhesive capsulitis. Prior studies using PCTMA in shoulder conditions have focused primarily on rotator cuff repair applications, and no previous randomized controlled trial has evaluated its use in the context of adhesive capsulitis.
